# Dimensionality reduction using singular vectors

**DOI:** 10.1038/s41598-021-83150-y

**Published:** 2021-02-15

**Authors:** Majid Afshar, Hamid Usefi

**Affiliations:** 1 Department of Computer Science, Memorial University of Newfoundland, St. John’s, NL Canada; 2 Department of Mathematics ans Statistics, Memorial University of Newfoundland, St. John’s, NL Canada

**Keywords:** Cancer, Computational biology and bioinformatics, Genetics, Mathematics and computing

## Abstract

A common problem in machine learning and pattern recognition is the process of identifying the most relevant features, specifically in dealing with high-dimensional datasets in bioinformatics. In this paper, we propose a new feature selection method, called Singular-Vectors Feature Selection (SVFS). Let $D= [A \mid \mathbf {b}]$ be a labeled dataset, where $\mathbf {b}$ is the class label and features (attributes) are columns of matrix *A*. We show that the signature matrix $S_A=I-A^{\dagger }A$ can be used to partition the columns of *A* into clusters so that columns in a cluster correlate only with the columns in the same cluster. In the first step, SVFS uses the signature matrix $S_D$ of *D* to find the cluster that contains $\mathbf {b}$. We reduce the size of *A* by discarding features in the other clusters as irrelevant features. In the next step, SVFS uses the signature matrix $S_A$ of reduced *A* to partition the remaining features into clusters and choose the most important features from each cluster. Even though SVFS works perfectly on synthetic datasets, comprehensive experiments on real world benchmark and genomic datasets shows that SVFS exhibits overall superior performance compared to the state-of-the-art feature selection methods in terms of accuracy, running time, and memory usage. A Python implementation of SVFS along with the datasets used in this paper are available at https://github.com/Majid1292/SVFS.

## Introduction

With the extraordinary advancements in high throughput gene expression profiling and DNA sequencing technologies, we are presented with the challenge of interpreting high-dimensional datasets. Nonetheless, this presents an opportunity for discovery of biological biomarkers that in turn can help for early detection of disease^1^ and identification of predictive and prognostic factors in disease management^2^. Genome-wide association studies (GWAS) can be performed on single-nucleotide polymorphism (SNP) arrays to identifying associations between loci and traits. Even though GWAS are proved to be useful^3^, there are some drawbacks as well. GWAS identifies loci so that each locus is statically significant (on its own). However, complex diseases are extremely polygenic and it therefore important to identify a subset of SNPs or genes that cumulatively explain the disease. Furthermore, most GWA studies require thousands of samples which can pose as a significant challenge.

Feature selection (FS) is another alternative for biomarker discovery. FS involves filtering and determining the relevant features from numerous irrelevant and redundant features, so FS can decrease the learning costs and improve the classification performance in many applications such as genomic data and remote sensing by turning the high-dimensional data into a lower dimension^4^. Features can be embedded into a lower-dimensional subspace in which different patterns appear to be considerably distinct with lower cost^5^. The importance of using FS methods on genomic data to supplement and improve the process of disease diagnosis is gaining increasing attention^6–9^. Hikichi et al.^10^ applied a correlation-centered approach and proposed a set of 12 predictive genes to diagnose cancer metastasis; their selected genes showed higher performance compared to the 76 genes previously reported by Wang et al.^11^. Recently, Jiang et al.^12^ applied a hybrid FS method for analyzing Endometrial Cancer data. In another study^13^, the authors focused on colon cancer and applied a hybrid FS method to obtain the optimal subset of genes using two independent datasets. Among 17,814 genes in the original dataset, 6 top relevant genes were selected in two phases. An independent dataset of colon cancer was used to validate the selected genes, resulting in 99.9% classification accuracy. Shukla et al.^14^ present a gene expression analysis on lymphoma cancer using several FS methods. Their experimental results showed that the highest classification accuracy is achieved using the top 20 selected genes. In a recent study, Sun et al.^15^ worked on high-dimensional microarray datasets and filtered data using the ReliefF method^16^ to reduce the dimensionality of gene expression data and then applied a modified Ant Colony Optimization algorithm^17^ to find the optimal subset of genes for colon, leukemia, lung, prostate, and brain cancers.

In this paper, we propose a new FS method based on singular vectors (SVFS). Let $D=[A \mid \mathbf{b} ]$ be a dataset, where *A* is an $m \times n$ matrix with *m* instances and *n* features, and $\mathbf{b}$ is the class label. We define the signature matrix $S_A$ of *A* by setting $S_A=I-A^{\dagger }A$, where $A^{\dagger }$ is the pseudo-inverse of *A*. We introduce a two-step irrelevant features filtering that maps the given dataset into a lower-dimensional subspace that includes less noisy and more informative features. Using the signature matrix $S_A$, features that have correlations to each other are clustered. The most important features are then picked from each cluster. This process can be optimized using two thresholds to make our model capable of handling a wide range of high dimensional data types. We view the data and interactions between all features globally in the sense that we measure the relevancy of features to $\mathbf {b}$ all at once and then breakdown the original feature space into a collection of lower dimensional subspaces. In contrast, many FS methods apply one or two discriminative concepts locally and at the individual feature level to obtain the most important features. Thus, they may perform well on some types of datasets and have inferior performances on other types of datasets. For example, as we shall see in Section 4, Fisher score^21^ and Trace ratio criterion^22^ have a good performances on biological benchmark datasets while they produce weak results on the image benchmark datasets.

We show in Section 3, that $S_A$ is the same as the orthogonal projection *P* onto the null space of *A*; hence *S* or *P* can be constructed using right singular vectors. We define a graph *G* where the nodes are columns of *A* and there is an edge between columns ${{\mathbf {F}}}_i$ and ${{\mathbf {F}}}_j$ if and only if $S_{i,j}\ne 0$. As we shall explain, each connected component of *G* corresponds to a subset of columns of *A* that are linearly dependent. In other words, the correlations between columns of *A* are encoded in the signature matrix $S_A$.

We view *D* as a matrix and form the signature matrix $S_D=I-D^{\dagger }D$. The cluster of *D* containing $\mathbf {b}$ consists of relevant features to $\mathbf {b}$ and all features in the other clusters are considered irrelevant. After removing irrelevant features, we update *A* and use the graph associated to $S_A$ to find the clusters. There are many efficient algorithms to find the clusters of a graph. We use Breadth-First Search (BFS)^18^ to find the features which are directly or indirectly connected to the other features. The novelty of our method is to use the signature matrix $S_D$ of *D* to detect and remove irrelevant features and then use the signature matrix $S_A$ of the reduced matrix *A* to partition the columns of *A* into clusters so that columns within a cluster correlate only with columns within the same cluster. Finally, we rank the features in a cluster based on the entries on the main diagonal of $S_A$ and select a small subset of top ranked features with the highest Mutual Information (MI) with respect to $\mathbf{b}$.

In order to evaluate the performance and efficiency of our method, we compare it with the state-of-the-art FS methods, namely Conditional Infomax Feature Extraction (CIFE)^19^, Joint Mutual Information (JMI)^20^, Fisher score^21^, Trace ratio criterion^22^, Least angle regression (LARS)^23^, Hilbert-Schmidt independence criterion least absolute shrinkage and selection operator (HSIC-Lasso)^24^, Conditional Covariance Minimization (CCM)^25^, and Sparse Multinomial Naive Bayes (SMNB)^26^ on a series of high dimensional benchmark as well as biological datasets.

The rest of this paper is structured as follows. An overview of the existing FS approaches is given in section 2. Then, in Section 3, we give a theoretical background along with some examples on synthetic data to show how our method removes irrelevant features and finds correlations between the rest of the features using the signature matrix *S*. Section 4 gives an account on specifications of the datasets and reports our experiment results. Finally, we provide a summary in Section 5.

## Related work

FS methods are categorized as filter, wrapper, and embedded methods^27^. The filter methods use some underlying and intrinsic properties of the features measured via univariate statistics, while the wrapper methods measure the importance of features based on the classifier performances. While optimizing the classifier performance is the essential goal of FS, and the wrapper methods have their own efficient internal classifiers, these methods are computationally more expensive in comparison with the filter methods due to the iterated learning steps of the wrapper methods and their cross-validation to avoid the risk of overfitting the model. The embedded methods are similar to the wrapper methods; however, the former mainly uses an intrinsic model building metric during the learning process.

Many FS algorithms work based on information-theoretical approaches which utilize various criteria to measure and rank the importance of features. The basic idea behind many information-theoretic methods is to maximize feature relevance and minimize feature redundancy^21^. Since feature correlation with class labels normally measures the relevance of the feature, most algorithms in this group are applied in a supervised manner. A brief introduction to basic information-theoretic concepts is given here.

Shannon entropy, as the primary measurement in information-theoretical approaches, measures the uncertainty of a discrete random variable. The entropy of a discrete random variable *X* is described as below:

1 $$\begin{aligned} H(X)=-\sum _{x_i \in X} P(x_i)log(P(x_i)), \end{aligned}$$

where $x_i$ is a specific value of *X* and $P(x_i )$ refers to the probability of $x_i$ over all values of *X*.

Second concept is the conditional entropy of *X* and *Y*,  which is another discrete random variable, defined as follows:

2 $$\begin{aligned} H(X|Y)=-\sum _{y_i \in Y} P(y_i) \sum _{x_i \in X} P(x_i | y_i)log(P(x_i | y_i)) \end{aligned}$$

where $P(y_i )$ is the prior probability of $y_i, P (x_i |y_j)$ refers to the conditional probability of $x_i$ and $y_j$.

To measure the amount of information shared between *X* and *Y*, MI or information gain is used, which is defined as follows:

3 $$\begin{aligned} I(X;Y)=H(X)-H(X|Y)=\sum _{x_i \in X} \sum _{y_i \in Y} P(x_i, y_i)log\left( \frac{P(x_i, y_i)}{P(x_i)P(y_i)}\right) \end{aligned}$$

where $P(x_i,y_j)$ is the joint probability of $x_i$ and $y_j$. MI is symmetric such that $I (X;Y ) = I (Y;X)$ and in case *X* and *Y* are independent, their MI would is zero. Since we applied the MI concept in our proposed method, two representative algorithms of information-theoretical based family are selected for comparison, including Conditional Infomax Feature Extraction (CIFE)^19^, Joint Mutual Information (JMI)^20^.

Several studies including CIFE^19^ and^28,29^ are based on the idea that the conditional redundancy between unselected features and selected features given class labels should be maximized rather than minimizing the feature redundancy. Minimum Redundancy Maximum Relevance (MRMR) reduces feature redundancy in the feature selection process. In contrast, JMI^20,30^ is introduced to increase the MI that is distributed between selected features and unselected features. There have been some improvements of JMI, see^31^.

Another category of FS methods is the similarity-based approaches that measure the feature relevances by their ability to preserve data similarities. The two superior similarity-based methods, i.e. the Fisher score^21^ and Trace Ratio criterion^22^ are selected to provide a basis for comparison with our proposed method.

Fisher score is a supervised feature selection method that explores features with high discriminant capacity. For sample points in different classes, Fisher score aims to maximize distances between samples; in contrast, it minimizes the distances between sample points in the same class. Trace Ratio criterion has the same idea of maximizing data similarity between-class of instances, while minimizing data similarity the within-class of instances. It computes a Trace Ratio norm by building two affinity matrices $S_w$ and $S_b$ to designate within-class and between-class data similarity.

Some approaches use aggregated sample data to select and rank the features^23,24,32,33^. The least absolute shrinkage and selection operator (LASSO) is an estimation method in linear methods that performs two main tasks: regularization and feature selection. For the first task, it calculates the sum of the absolute values of the model parameters, and the sum must be less than a prefixed upper bound. Therefore, by applying a regularization (shrinking) process, it penalizes the coefficients of the regression variables shrinking, some of them are set to zero. For the second task, the features that still have a non-zero coefficient after the regularization process are chosen to be part of the model. The goal of this process is to lessen the prediction error.

Least angle regression (LARS) proposed by Efron et al.^23^ works based on LASSO and is a linear regression method that computes all least absolute shrinkage and selection operator^33^ estimates and selects those features which are highly correlated to the already selected ones. Yamada et al. in^24^ proposed a non-linear FS method for high-dimensional datasets called Hilbert-Schmidt independence criterion least absolute shrinkage and selection operator (HSIC-Lasso). By solving a Lasso problem and using a set of kernel functions, HSIC-Lasso selects informative non-redundant features. In another work^34^ called Least Angle Nonlinear Distributed (LAND), the authors have improved the computational power of the HSIC-Lasso. They illustrated through comprehensive examinations that LAND and HSIC-Lasso achieve comparable classification accuracies and dimension reduction. However, LAND has the advantage that it can be developed on parallel distributed computing.

HSIC-Lasso and LAND are based on a convex optimization problem with a $\ell _1$-norm penalty on the regression coefficients to improve sparsity while having a significantly high computational cost, especially on high dimensional data. Very recently, Askari et al.^26^ proposed a sparse version of naive Bayes, leading to a combinatorial maximum likelihood capable of solving the binary data and providing explicit bounds on the duality gap for multinomial data, at a fraction of the computing cost.

We also remark that FS is applied and used in various domains including gene selection, face recognition, handwriting identification, and remote sensing^35–38^.

## Proposed approach

Let *A* be an $m\times n$ matrix of rank $\rho$ and consider the singular value decomposition (SVD) of *A* as $A=U\Sigma V^T$, where $U_{m\times m}$ and $V_{n\times n}$ are orthogonal matrices and $\Sigma =\text {diag}(\sigma _1, \ldots , \sigma _{\rho }, 0, \ldots , 0 )$ is an $m\times n$ diagonal matrix. We denote column *j* of *V* by $\mathbf {v}_j$ and row *j* of *V* by $\mathbf {v}^j$. Furthermore, we partition $\mathbf {v}^j$ as $$\mathbf {v}^j= \left[ \begin{array}{c|c} \mathbf {v}^{j,1}&\mathbf {v}^{j,2} \end{array} \right] $$, where $\mathbf {v}^{j,1}$ consists of the first $\rho$ entries of $\mathbf {v}^{j}$ and $\mathbf {v}^{j,2}$ is the remaining $n-\rho$ entries. Note that $A\mathbf {v}_j=0$, for all $\rho +1\le j\le n$, and moreover $\ker (A)$ is spanned by all $\mathbf {v}_{\rho +1}, \ldots , \mathbf {v}_{n}$. We denote by ${{\mathbf {F}}}_j$ the *j*-th column of *A*.

Let ${\bar{V}}$ be the matrix consisting of columns $\rho +1, \ldots , n$ of *V*, that is $${\bar{V}}= \left[ \begin{array}{c|c|c} \mathbf {v}_{\rho +1}&\cdots&\mathbf {v}_{n} \end{array} \right] . $$ Let $P={\bar{V}} \bar{V}^T$. Note that $P\mathbf {w}=\mathbf {w}$, for every $\mathbf {w}\in {\mathcal {N}}(A)$, where ${\mathcal {N}}(A)$ is the null space of *A*. Indeed, *P* is the orthogonal projection onto ${\mathcal {N}}(A)$, that is range of *P* is ${\mathcal {N}}(A)$, $P^2=P$ and $P^T=P$. We also let $S=I-A^\dagger A$. By Lemma 2.1 in^39^, we know that *S* and *P* are indeed the same. Nevertheless, the computational complexity of computing of *S* and *P* might be different. For to compute *P* we just need the right singular vectors of the symmetric matrix $A^TA$. On the other hand, if *A* is full row rank then we know $A^{\dagger }=A^T(AA^T)^{-1}$. So in case *A* has full row-rank, the complexity of computing *S* is the same as complexity of matrix inversion.

Let $D=[A\mid \mathbf {b}]$ be a dataset, say a binary Cancer dataset, where rows of *A* are samples (patients), columns of *A* are features (gene expressions) and $\mathbf {b}$ is the class label that each of its entries are either 0 (noncancerous) or 1 (cancerous). In large datasets that are a large number of features that are irrelevant. For example, in gene expression datasets, there are a large number of genes that are not expressed. So, identifying and removing features that have negligible correlation with the class labels is crucial. The aim of FS is to come up with a minimal subset of features that can be used to predict the class labels as accurate as possible. There might be redundancies (correlations) among relevant features that must be detected and removed.

As we explain below, we use the matrix S (or P) to divide the set of all features into clusters where features within a cluster correlate with each other and different clusters are linearly independent from each other. So, a set of linear dependencies defines the correlations within a cluster.

Without loss of generality, we assume that $\{{{\mathbf {F}}}_1, \ldots ,{{\mathbf {F}}}_t\}$ is a cluster, that is ${{\mathbf {F}}}_1, \ldots ,{{\mathbf {F}}}_t$ are linearly dependent and independent of the rest of the ${{\mathbf {F}}}_k$, where $k\ge t+1$. The following theorem from^39^, is the first major step to identify clusters.

### **Theorem 1**

*Suppose that* $\{{{\mathbf {F}}}_1, \ldots ,{{\mathbf {F}}}_t\}$ *is a cluster. Then* $P_{i,j}=0$, *for every* $1\le j\le t$ *and every* $i\ge t+1$.

### *Example 1*

Consider a $100\times 80$ synthetic matrix *A* with the only relations between columns of *A* as follows:

$$\begin{aligned} \begin{array}{cccc} -{{\mathbf {F}}}_1+3{{\mathbf {F}}}_2+6{{\mathbf {F}}}_4=0,&{} -{{\mathbf {F}}}_6-2{{\mathbf {F}}}_{10}+2{{\mathbf {F}}}_5-4{{\mathbf {F}}}_{11}=0,&{} -{{\mathbf {F}}}_3-6{{\mathbf {F}}}_2+3{{\mathbf {F}}}_4=0,\\ -{{\mathbf {F}}}_7-{{\mathbf {F}}}_{10}-3{{\mathbf {F}}}_{11}=0,&{} -{{\mathbf {F}}}_5+3{{\mathbf {F}}}_{11}+{{\mathbf {F}}}_{10}=0,&{} -{{\mathbf {F}}}_{8}+3{{\mathbf {F}}}_{10}+2{{\mathbf {F}}}_{11}=0,&{} -{{\mathbf {F}}}_9+5{{\mathbf {F}}}_5-{{\mathbf {F}}}_7=0. \end{array} \end{aligned}$$

The signature matrix $S_A$ (rounded up to two decimals) is:

4 $$\begin{aligned} \left( \begin{array}{cccccccccccccc} 0.02 &{} -0.07 &{} 0 &{} -0.13 &{} 0 &{} 0 &{} 0 &{} 0 &{} 0 &{} 0 &{} 0 &{} 0&{} \cdots &{} 0\\ -0.07 &{} 0.98 &{} 0.13 &{} 0 &{} 0 &{} 0 &{} 0 &{} 0 &{} 0 &{} 0 &{} 0 &{} 0&{} \cdots &{} 0\\ 0 &{} 0.13 &{} 0.02 &{} -0.07 &{} 0 &{} 0 &{} 0 &{} 0 &{} 0 &{} 0 &{} 0 &{} 0&{} \cdots &{} 0\\ -0.13 &{} 0 &{} -0.07 &{} 0.98 &{} 0 &{} 0 &{} 0 &{} 0 &{} 0 &{} 0 &{} 0 &{} 0&{} \cdots &{} 0\\ 0 &{} 0 &{} 0 &{} 0 &{} 0.97 &{} 0 &{} 0.03 &{} 0 &{} -0.16 &{} 0.01 &{} -0.01 &{} 0&{} \cdots &{} 0\\ 0 &{} 0 &{} 0 &{} 0 &{} 0 &{} 0.37 &{} 0 &{} -0.44 &{} 0 &{} -0.19 &{} 0.06 &{} 0&{} \cdots &{} 0\\ 0 &{} 0 &{} 0 &{} 0 &{} 0.03 &{} 0 &{} 0.97 &{} 0 &{} 0.16 &{} -0.01 &{} 0.01 &{} 0&{} \cdots &{} 0\\ 0 &{} 0 &{} 0 &{} 0 &{} 0 &{} -0.44 &{} 0 &{} 0.69 &{} -0.03 &{} -0.13 &{} 0.04 &{} 0&{} \cdots &{} 0\\ 0 &{} 0 &{} 0 &{} 0 &{} -0.16 &{} 0 &{} 0.16 &{} -0.03 &{} 0.06 &{} 0.03 &{} -0.06 &{} 0&{} \cdots &{} 0\\ 0 &{} 0 &{} 0 &{} 0 &{} 0.01 &{} -0.19 &{} -0.01 &{} -0.13 &{} 0.03 &{} 0.94 &{} 0.02 &{} 0&{} \cdots &{} 0\\ 0 &{} 0 &{} 0 &{} 0 &{} -0.01 &{} 0.06 &{} 0.01 &{} 0.04 &{} -0.06 &{} 0.02 &{} 0.99 &{} 0&{} \cdots &{} 0\\ 0 &{} 0 &{} 0 &{} 0 &{} 0 &{} 0 &{} 0 &{} 0 &{} 0 &{} 0 &{} 0 &{} 0 &{} \cdots &{} 0\\ \vdots &{} \vdots &{}\vdots &{}\vdots &{}\vdots &{}\vdots &{}\vdots &{}\vdots &{}\vdots &{}\vdots &{}\vdots &{}\vdots &{}\cdots &{}\vdots \\ 0 &{} 0 &{} 0 &{} 0 &{} 0 &{} 0 &{} 0 &{} 0 &{} 0 &{} 0 &{} 0 &{} 0&{} \cdots &{} 0 \\ \end{array}\right) \end{aligned}$$

We note that *A* is randomly generated and the only constrain on *A* is the set of dependent relations given above. We can see that *S* has a block diagonal form, where each block corresponds to a cluster. So, features ${{\mathbf {F}}}_1, \ldots , {{\mathbf {F}}}_4$ constitute a cluster. Similarly, $\{{{\mathbf {F}}}_5, \ldots , {{\mathbf {F}}}_{11}\}$ is another cluster. Note that $\{{{\mathbf {F}}}_i\}$ is a singleton cluster, for all $i\ge 12$. We provide some details about these facts in the next lemma.

### **Lemma 1**

*Let A be the matrix in Example* 1. *Then,* $P_{i,j}=0$ *for all* $1\le i\le 4$ *and* $5\le j\le n$.

### *Proof*

We note that rank of *A* is $\rho =73$. Hence, $A{{\mathbf {v}}}_k=0$, for every $74\le k\le 80$. Since $A{{\mathbf {v}}}_k=0$ yields a dependence relation between columns of *A* and ${{\mathbf {F}}}_1, \ldots ,{{\mathbf {F}}}_4$ are independent from the rest of the columns, we deduce that $A{\bar{{{\mathbf {v}}}}}_k=0$, where ${\bar{{{\mathbf {v}}}}}_k$ consists of the first 4 entries of ${{\mathbf {v}}}_k$. Then we form the matrix $$ M= \left[ \begin{array}{c|c|c} \bar{{{\mathbf {v}}}}_{74}&\cdots&\bar{{{\mathbf {v}}}}_{80} \end{array} \right] $$. Since any linear combination of columns of *M* provides a dependence relation between ${{\mathbf {F}}}_1, \ldots ,{{\mathbf {F}}}_4$, we can use elementary (column) operations to transform *M* into the matrix ${\bar{C}}_1$:

$$\begin{aligned} {\bar{C}}_1= \left( \begin{array}{cccccc} -1.0 &{} 0 &{}0&{}0&{}0&{}0\\ 0 &{} -1.0&{}0&{}0&{}0&{}0\\ -0.5 &{} -0.17&{}0&{}0&{}0&{}0\\ 7.5 &{} 0.5&{}0&{}0&{}0&{}0\\ \end{array}\right) . \end{aligned}$$

Then $$\left[ \begin{array}{c|c|c} {{\mathbf {F}}}_1&\cdots&{{\mathbf {F}}}_4 \end{array} \right] {\bar{C}}_1=0$$; in other words columns of ${\bar{C}}_1$ give us the minimal relations between ${{\mathbf {F}}}_1, \ldots ,{{\mathbf {F}}}_4$. Let *k* be in the range $74\le k\le 80$. Since $A {{\mathbf {v}}}_k=0$, we have $v_{1,k}{{\mathbf {F}}}_1+v_{2,k}{{\mathbf {F}}}_2+v_{3,k}{{\mathbf {F}}}_3+v_{4,k}{{\mathbf {F}}}_4=0$. Substituting for ${{\mathbf {F}}}_1$ and ${{\mathbf {F}}}_2$ in terms of ${{\mathbf {F}}}_3$ and ${{\mathbf {F}}}_4$ using the matrix ${\bar{C}}_1$, we get

$$\begin{aligned} v_{1,k}(-0.5{{\mathbf {F}}}_3+7.5{{\mathbf {F}}}_4)+v_{2,k}(-\frac{1}{6}{{\mathbf {F}}}_3+0.5{{\mathbf {F}}}_4)+v_{3,k}{{\mathbf {F}}}_3+v_{4,k}{{\mathbf {F}}}_4=0. \end{aligned}$$

We deduce that

$$\begin{aligned} -0.5v_{1,k}-\frac{1}{6}v_{2,k}+v_{3,k}=0,\quad&7.5v_{1,k}+0.5v_{2,k}+v_{4,k}=0. \end{aligned}$$

Since the above equations hold for every *k* in the range $\rho +1\le k\le n$, we deduce that

$$\begin{aligned} -0.5{\mathbf {v}}^{1,2}-\frac{1}{6}{{\mathbf {v}}}^{2,2}+\mathbf {v}^{3,2}=0,\quad&7.5{\mathbf {v}}^{1,2}+0.5{{\mathbf {v}}}^{2,2}+\mathbf {v}^{4,2}=0. \end{aligned}$$

Let *j* be in the range $5\le j\le n$. Then taking the dot product with $\mathbf {v}^{j,2}$ yields

5 $$\begin{aligned} 0.5P_{1,j}-\frac{1}{6}P_{2,j}+P_{3,j}=0,\qquad&7.5P_{1,j}+0.5P_{2,j}+P_{4,j}=0. \end{aligned}$$

Let $$ C=\left[ \begin{array}{c|c} {\bar{C}}_1 &{}0\\ \hline 0 &{}0 \end{array} \right] $$ be an $n\times n$ matrix. Let $\mathbf {c}_1, \ldots , \mathbf {c}_n$ be the columns of *C* and denote by $\mathbf {p}^j$ the *j*-th row of *P*. Since $P\mathbf {c}_i=\mathbf {c}_i$, we deduce that $\mathbf {p}^j\mathbf {c}_i=\mathbf {c}_{i,j}=0$, since $j\ge 5$. Hence,

6 $$\begin{aligned} -P_{1,j}-0.5P_{3,j}+7.5P_{4,j}=0,\quad&-P_{2,j}-\frac{1}{6}P_{3,j}+0.5P_{4,j}=0. \end{aligned}$$

Putting together the Equations (5) and (6), we deduce that

$$\begin{aligned} B\begin{bmatrix} P_{1,j}&P_{2,j}&P_{3,j}&P_{4,j} \end{bmatrix}^T =0, \end{aligned}$$

where

$$\begin{aligned} B= \begin{bmatrix} -1 &{} 0&{} -0.5&{} 7.5 \\ 0&{}-1 &{} -0.17&{} 0.5 \\ -0.5&{} -0.17&{}1 &{} 0 \\ 7.5 &{} 0.5&{} 0&{}1 \\ \end{bmatrix} =\left[ \begin{array}{c|c} -I_2 &{}Z^T\\ \hline Z &{}I_2 \end{array} \right] , \quad Z= \left[ \begin{array}{cccc} -0.5 &{} -0.17\\ 7.5 &{} 0.5 \end{array} \right] . \end{aligned}$$

Since, by Lemma 2.4 in^39^, *B* is invertible, we deduce that $P_{1,j} = \cdots = P_{4,j}=0$.

$$\square $$

In general it follows from Theorem 1 that after re-ordering the columns of *A*, the matrix *S* has a block-diagonal form where each block corresponds to a cluster. Of course, a priori, columns within the same cluster are not next to each other in the matrix *A*. Furthermore, the converse of Theorem 1 is not true in general. In other words, $P_{i,j}$ could be zero even when ${{\mathbf {F}}}_i$ and $F_j$ are in the same cluster as can be seen in Example 1 where $P_{1,3}=P_{5,6}=0$.

**Figure 1:**
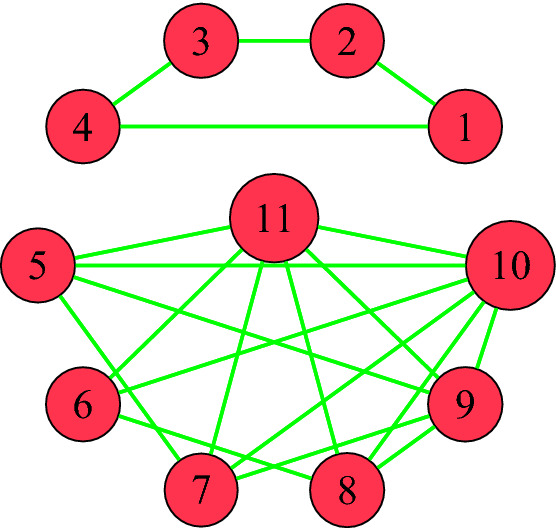
The graph associated to matrix *A* demonstrating the two clusters.

To find the clusters, we define a graph *G* whose vertices consists of ${{\mathbf {F}}}_1, \ldots , {{\mathbf {F}}}_n$ and we define an edge between ${{\mathbf {F}}}_i$ and ${{\mathbf {F}}}_j$ if and only if $P_{i, j}\ne 0$. The graph associated to matrix *A* in Example 1 is depicted in Figure 1.

Even though, there may not be an edge between two nodes of the same cluster, it turns out there is always a path connecting every two nodes in the same cluster. This fact which is Theorem 2.10 in^39^, can be summarized as follows.

### **Theorem 2**

*The sub-graph of* *G* *consisting of nodes* ${{\mathbf {F}}}_1,\ldots , {{\mathbf {F}}}_t$ *and corresponding edges is connected.*

As we mentioned, in real datasets there are many irrelevant features. To identify the irrelevants, we construct the signature matrix $S_D$ of *D* and identify the cluster that includes $\mathbf {b}$. The remaining clusters consist of features that have a negligible correlation with $\mathbf {b}$. So, we can remove all other clusters from *A*.

### *Example 2*

Let *A* be the synthetic matrix as in Example 1 and $\mathbf {b}= {{\mathbf {F}}}_1-3{{\mathbf {F}}}_3+2{{\mathbf {F}}}_9-{{\mathbf {F}}}_{14}$. The last row of signature matrix $S_D$ (rounded up to four decimals) is:

$$\begin{aligned} \left(\begin{array}{llllllllllllllllllll} -0.0364 & -0.0170 & 0.1093 & 0.0024 & -0.0234 & 0.0006 &0.0234 & -0.0043 & -0.1403 & 0.0049& -0.0094 & 0&0&&0.0373& 0&\ldots&0 & 0.0373\\ \end{array}\right) \end{aligned}$$

The cluster containing $\mathbf {b}$ consists of features ${{\mathbf {F}}}_i$ such that $S_{i,n+1}\ne 0$. So, we identify the columns ${{\mathbf {F}}}_j$ where $j= 12, 13$ or $15 \le j \le 100$ as irrelevant features and remove them from *A*.

Alternatively, we can also identify irrelevant features by looking at the least-squares solutions of the system $A\mathbf {x}=\mathbf {b}$. Note that $\mathbf {x}=A^\dagger \mathbf {b}$, where $A^\dagger$ is the pseudo-inverse of *A*. Each component $x_i$ of $\mathbf{x}$ can be considered as an assigned weight to the feature ${{\mathbf {F}}}_i$ of *A*. Hence, the bigger the $|x_i|$, the more salient ${{\mathbf {F}}}_i$ is in correlation with $\mathbf {b}$.

### *Example 3*

Let *A* be the synthetic matrix as in Example 1 and $\mathbf {b}= {{\mathbf {F}}}_1-3{{\mathbf {F}}}_3+2{{\mathbf {F}}}_9-{{\mathbf {F}}}_{14}$. We solve $A\mathbf {x}=\mathbf {b}$ using the least-squares method where the vector $\mathbf {x}$ (rounded up to two decimals) is:

$$\begin{aligned}\left(\begin{array}{llllllllllllllllllll} 0.98&0.46&-2.93&-0.07&0.63&-0.02&-0.63&0.11&3.77&-0.13&0.25&0&0&-1&0&\ldots&0\\ \end{array}\right)\end{aligned}$$

Let $\mathbf {x}=[x_1, \ldots , x_n]$, where each $x_i$ is an assigned weight to ${{\mathbf {F}}}_i$. Hence, we can approximate $\mathbf {b}$ as a linear combination of the form $x_1{{\mathbf {F}}}_1+ \cdots + x_n{{\mathbf {F}}}_n$. Therefore, $x_i=0$ implies ${{\mathbf {F}}}_i$ has no impact on $\mathbf {b}$ and that ${{\mathbf {F}}}_i$ is irrelevant. According to vector $\mathbf {x}$, $x_i=0$ for $i=12, 13$ and $15 \le i \le n$ and we remove the corresponding ${{\mathbf {F}}}_i$ from *A*.

Since, the notion of relevancy is not quantitative and one has to be cautious in removing features, we set a soft threshold $Th_{irr}$ and incorporate both the methods explained in Examples 2 and 3. In this paper, we first filter out features with minimal weight, that is features with $|x_i|$ less than $\frac{1}{n} \sum _{i=1}^n |x_i| \times Th_{irr}$ where $\frac{1}{n} \sum _{i=1}^n |x_i|$ is the average of the $|x_i|$s. Then we set $|P_{i, n+1}|=0$ whenever $|P_{i, n+1}|< Th_{irr}$. Note that the last row of $S_D$ reflects the correlations with $\mathbf {b}$. We sort the last row of $S_D$ as descending and remove the features outside the length of $\frac{1}{n} \sum _{i=1}^n |P_{i, n+1}| \times (Th_{irr} +1)$. So, we apply a two-step process with a soft threshold at each step to remove the irrelevant features. Note that we still denote by *A* the reduced matrix obtained after removing the irrelevant features.

In the next step, we identify redundant features. To do so, we use the signature matrix $S_A$ of *A* and consider the associated graph. There are many efficient algorithms to find the clusters or connected components of a graph. One such algorithm is Breadth-First Search (BFS)^18^. By applying the BFS starting from vertex $\mathbf{F} _i$, we can determine its accessible vertices. In other words, different clusters can be specified using BFS on the unvisited vertex $\mathbf{F} _i$. For example, in Fig. 1, the first unvisited vertex (feature) is $\mathbf{F} _1$, and applying BFS on $\mathbf{F} _1$ would visit $\mathbf{F} _2, \mathbf{F} _4, \mathbf{F} _3$, respectively. Since there is no unvisited connected feature, the first cluster consists of $\mathbf{F} _1$ to $\mathbf{F} _4$. Then, BFS should be applied to the next unvisited $\mathbf{F} _i$, and add the consequently visited features to the next cluster until all the connected vertices in the current cluster are visited.

From each resulting cluster, a feature that carries the highest MI with $\mathbf{b}$ is selected as the output of the SVFS method. The selected feature from each cluster is, indeed, the one that best represents that cluster. In real datasets we might inherently encounter minor correlations between features, that is in the matrix $S_A$ we might see very small entries that indicate weak correlations. We use a threshold $Th_{red}$ to map the weak feature correlations to zero. Also, in case we encounter a few clusters with numerous vertices, we set a threshold $\alpha$ to split the clusters with more than $\alpha$ vertices into sub-clusters with the maximum of $\alpha$ vertices. The features in each sub-cluster are then sorted based on the last row of $S_D$, and the top $\beta$ features are selected to find their highest MI with $\mathbf{b}$. The choice of $\beta$ features in each sub-cluster is with the aim of reducing the computational cost of the MI calculations.

### Algorithm

**Figure 2:**
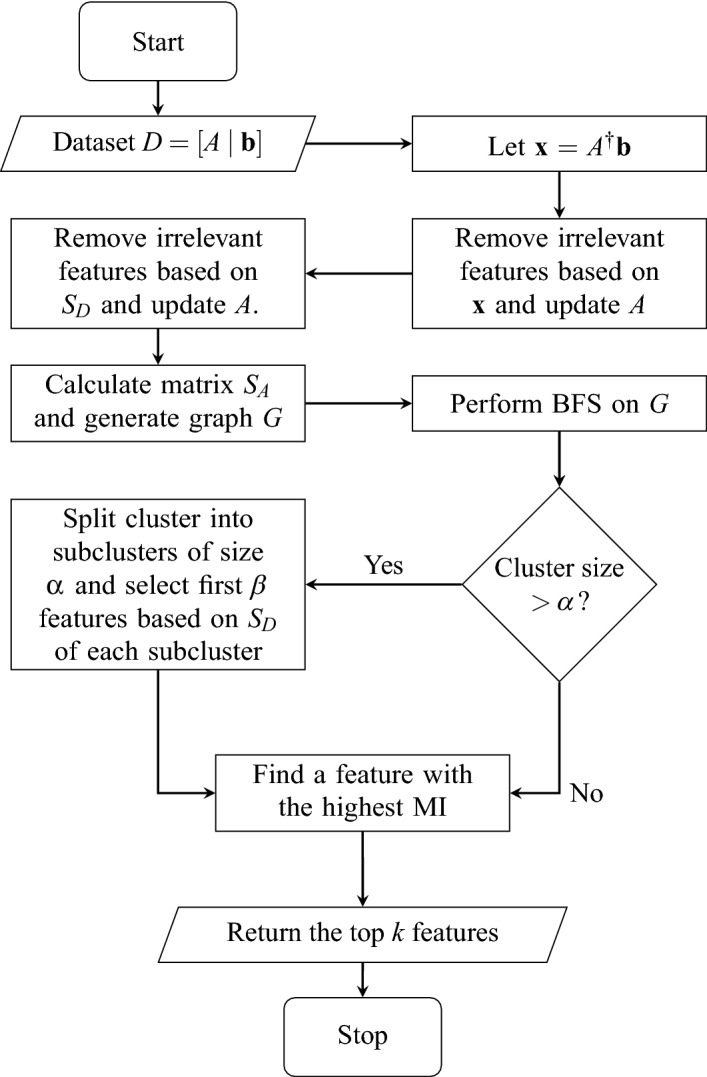
Flowchart of SVFS.

In this section, we present the algorithm and flowchart of SVFS in Figure 2.
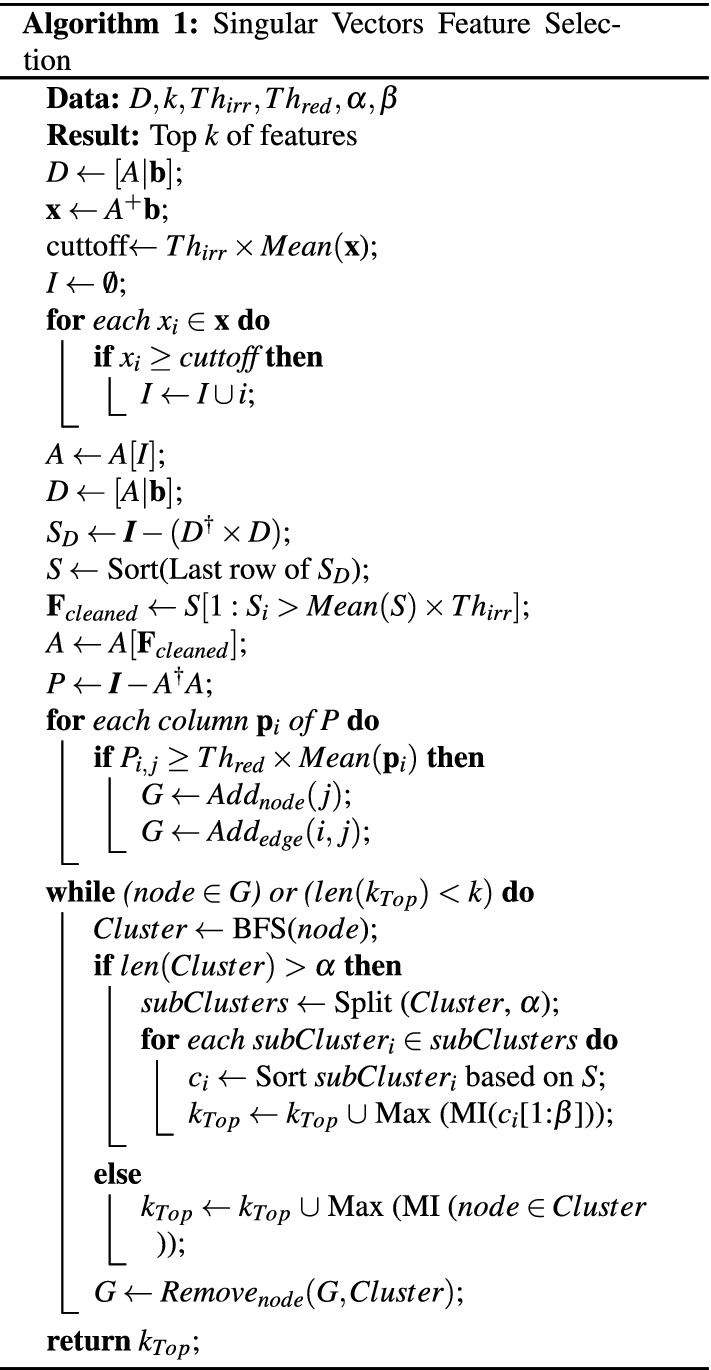


The while loop in the algorithm essentially demonstrates finding the connected components of the graph associated to *P*. The well-known BFS algorithm finds the connected components of a graph *G*(*V*, *E*) with complexity ${\mathcal {O}}(|V| +|E|)$. In our case, |*E*| is the number of non-zero entries in *P*. So, the worst case in the algorithm can happen when $|E|=\dfrac{n(n-1)}{2}$. Hence, the complexity of the while loop is ${\mathcal {O}}(n^2)$. We also mention that parallel algorithms for BFS have been of great interest, see for example^40^.

The complexity of computing $S=I-A^{\dagger }A$ is more delicate. There is extensive research on finding efficient and reliable methods to find $A^\dagger$, see for example^41–43^. One of the most commonly used methods is the Singular Value Decomposition (SVD) which is very accurate but time and memory intensive especially in the case of large matrices. The complexity of computing SVD of $A_{m\times n}$ is ${\mathcal {O}}(\min (mn^2,m^2n))$.

Pseudo-inverses are used in neural learning algorithms to solve large least square systems. So, there is a great interest in finding the pseudo-inverse efficiently. Courrieu in^44^ proposed an algorithm called Geninv based on Cholesky factorization and showed that the computation time is substantially shorter, particularly for large systems. It is noted in^44^ that the complexity of Geninv on a single-threaded processor is ${\mathcal {O}}(\min (m^3, n^3))$ whereas in a multi-threaded processor, the time complexity is ${\mathcal {O}}(\min (m,n))$. The authors in^45^ investigated the effective computation of the pseudo-inverse for neural networks and concluded that QR factorization with column pivoting along with Geninv works well. Since our implementation is single-threaded and $m<<n$, the complexity of pseudo-inverse is ${\mathcal {O}}(m^3)$. We can conclude that the complexity of our algorithm is at most ${\mathcal {O}}(\max (m^3, n^2))$.

## Experimental result

We compared our method with eight state-of-the-art FS methods including Conditional Infomax Feature Extraction (CIFE), Joint Mutual Information (JMI), Fisher score, Trace Ratio criterion, Least angle regression (LARS), Hilbert-Schmidt independence criterion least absolute shrinkage and selection operator (HSIC-Lasso), Conditional Covariance Minimization (CCM), and Sparse Multinomial Naive Bayes (SMNB). We used the scikit-feature library, which is an open-source feature selection repository in Python developed in the Arizona State University (ASU). It includes the implementation of CIFE, JMI, LARS, Fisher, and Trace Ratio methods. The reset of methods, namely, HSIC-Lasso, CCM, and SMNB are implemented in Python by their authors. To have a fair comparison among the different FS methods, we take advantage of 5-fold stratified cross-validation (CV) of the dataset so that 80% of each class is selected for FS. Then we use the Random Forest (RF) classifier with its default setting implemented in^46^, to build a model based on the selected features and evaluate the model on the remaining 20% of the dataset. We report the average classification accuracy over 10 independent runs (twice 5-fold CV) using the RF classifier on each dataset.

### Datasets

We selected a variety of publicly available datasets from two sources, i.e. Gene Expression Omnibus (GEO) which has various real genomic data, and the scikit-feature selection repository at Arizona State University which has benchmark biological and face image data to perform feature selection and classification. The specifications of these datasets are given in Tables 1 and 2.

**Table 1:** Benchmark Datasets Specifications.

Dataset	#Samples	#Features	Type	**#Labels**	Proportion of labels									
					**1**	**2**	**3**	**4**	**5**	**6**	**7**	**8**	**9**	**10**
TOX$\_$171	171	5,748	Biological	4	26.3%	26.3%	22.8%	24.6%	–	–	–	–	–	–
SMK$\_$CAN$\_$187	187	19,993	Biological	2	48.1%	51.9%	–	–	–	–	–	–	–	–
Prostate$\_$GE	102	5,966	Biological	2	49%	51%	–	–	–	–	–	–	–	–
lymphoma	96	4,026	Biological	9	47.9%	10.4%	9.4%	11.4%	6.3%	6.3%	4.1%	2.1%	2.1%	–
leukemia	72	7,070	Biological	2	65.3%	34.7%	–	–	–	–	–	–	–	–
lung	203	3,312	Biological	5	68.5%	8.4%	10.3%	9.8%	3%	–	–	–	–	–
GLIOMA	50	4,434	Biological	4	28%	14%	28%	30%	–	–	–	–	–	–
GLI$\_$85	85	22,283	Biological	2	30.6%	69.4%	–	–	–	–	–	–	–	–
CLL$\_$SUB$\_$111	111	11,340	Biological	3	9.9%	44.1%	46%	–	–	–	–	–	–	–
ALLAML	72	7,129	Biological	2	65.3%	34.7%	–	–	–	–	–	–	–	–
colon	62	2,000	Biological	2	64.5%	35.5%	–	–	–	–	–	–	–	–
NCI9	60	9,712	Biological	9	15%	15%	13.3%	8.3%	11.7%	10%	13.3%	10%	3.33%	–
pixraw10P	100	10,000	Image	10	10%	10%	10%	10%	10%	10%	10%	10%	10%	10%
warpAR10P	130	2,400	Image	10	10%	10%	10%	10%	10%	10%	10%	10%	10%	10%
warpPIE10P	210	2,420	Image	10	10%	10%	10%	10%	10%	10%	10%	10%	10%	10%
orlraws10P	100	10,304	Image	10	10%	10%	10%	10%	10%	10%	10%	10%	10%	10%

**Table 2:** Genomic Datasets Specifications.

Dataset	Samples	# Original F	# Cleaned F	# Labels	Proportion of labels			
					1	2	3	4
GDS1615	127	22,282	13,649	3	33%	20.5%	46.5%	–
GDS3268	202	44,290	29,916	2	36.1%	63.9%	–	–
GDS968	171	12,625	9,117	4	26.3%	26.3%	22.8%	24.6%
GDS531	173	12,625	9,392	2	20.8%	79.2%	–	–
GDS2545	171	12,625	9,391	4	10.6%	36.8%	38%	14.6%
GDS1962	180	54,675	29,185	4	12.8%	14.4%	45	27.8%
GDS3929	183	24,526	19,334	2	69.9%	30.1%	–	–
GDS2546	167	12,620	9,583	4	10.2%	35.3%	39.5%	15%
GDS2547	164	12,646	9,370	4	10.4%	35.4%	39%	15.2%

The pre-processing of GEO datasets used in this research was carried out by cleaning and converting the NCBI datasets to the CSV format. The mapping between the gene samples and the probe IDs has been retrieved using GEO2R^47^ and the probe IDs that did not have a gene mapping have been removed. For each gene, the expression values are obtained by averaging the expression values of all the probe IDs mapped to that specific gene. The k-Nearest Neighbors (kNN) imputation method was used to handle the missing values.

### Hardware and software

Our proposed method SVFS and other methods described in section 4 have been run on an IBM LSF 10.1.0.6 machine (Suite Edition: IBM Spectrum LSF Suite for HPC 10.2.0) with requested 8 nodes, 16 GB of RAM, and 8 GB swap memory using Python 3.6. Note that we only set 240 GB of RAM for the CCM model as it requires a high volume of memory.

### Parameters

The input parameters of our proposed SVFS method are $k, Th_{irr}, Th_{red}, \alpha , \beta$. The parameter *k* denotes the number of selected features and is a common parameter in all the methods evaluated in this study. There is no fixed procedure in the literature for determining the optimum value of k, but in many research works^48–51^, it is set to 50 which seems to be satisfactory in many cases. However, we take *k* in a wider range from 10 and 90 to ensure a fairground for comparison. When a subset of *k* features are returned as the output of a FS algorithm, we feed the first *t* features from the subset to the classifier to find an optimal *t* so that the subset of first *t* features yields the highest accuracy. This set up is applied across all FS methods. Also, we report average classification accuracy of a model over 10 independent runs (we run stratified 5-fold CV twice).

The parameter $Th_{irr}$ is the threshold set to filter out the irrelevant features. In this paper, we set the value of $Th_{irr}$ to 3. The parameter $Th_{red}$ is another threshold defined to deal with the low level of sparsity of *S*. In real-world large datasets, the condition $S_{i,j}=0$ might rarely be encountered. Indeed, the threshold $Th_{red}$ maps the weak feature correlations to zero. Here, we have set the value of $Th_{red}$ to 4 for the biological datasets and 7 for the face image datasets. The parameter $\alpha$ is used when facing big clusters to divide the clusters into subclusters with $\alpha$ members. The parameter $\beta$ is the number of features selected from each of the subclusters with $\alpha$ members. In this work, we have set the values of $\alpha$ and $\beta$ to 50 and 5, respectively.

### Results

**Figure 3:**
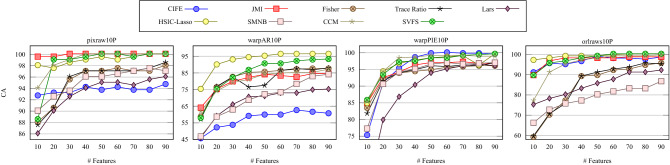
Average classification accuracy of feature selection by CIFE, JMI, Fisher, Trace Ratio, Lars, HSIC-Lasso, SMNB, CCM and SVFS over 10 runs on benchmark face image datasets.

The average classification accuracies over 10 independent runs (twice 5-fold CV) using the RF classifier on the datasets described in Section 4.1 are presented in this section. In Figure 3, we present the classification accuracy of SVFS compared to the other FS methods on 4 benchmark face image datasets. As it can be seen, our method attains either the best or second best accuracy compared to other FS methods. It is interesting to note that SVFS attains 100% accuracy on all of pixraw10P, warpPIE10P, and orlraws10P with at most 90 features.

**Figure 4:**
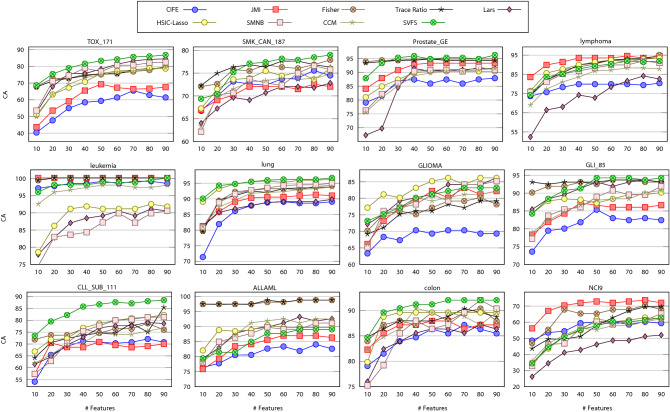
Average classification accuracy of feature selection by CIFE, JMI, Fisher, Trace Ratio, Lars, HSIC-Lasso, SMNB, CCM and SVFS over 10 independent runs on benchmark biological datasets.

Figure 4 shows the classification accuracy performance of SVFS compared to the other methods on benchmark biological datasets. As we can see, SVFS has performed consistently well and achieved the highest accuracy in 7 out of the 12 cases, while producing reasonably good accuracies in most of the other cases as well. JMI has produced the highest accuracy in 3 cases, where Fisher and HSIC-Lasso have shown their best performance in GLIOMA and ALLAML datasets, respectively. As we mentioned, the thresholds $Th_{irr}$ and $Th_{red}$ are set for 3 and 4, respectively for all biological datasets. However, it is possible to tune these thresholds and get better results. For example, if we set $Th_{irr}=1.2$ and $Th_{red}=2$, we get an average accuracy of 94.52 and 96.37 on ALLAML and Lymphoma datasets, respectively, and using at most 50 features ($\alpha =50, \beta =15$). Similarly, $Th_{irr}=1.1$ and $Th_{red}=2$, gives an average accuracy of 87 on GLIOMA dataset ($\alpha =50, \beta =15$), while $Th_{irr}=1.2$ and $Th_{red}=4$, gives an average accuracy of 74.14 on NCI9 dataset ($\alpha =50, \beta =10$).

**Figure 5:**
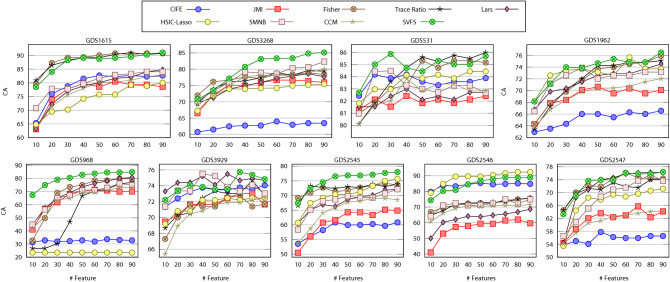
Average classification accuracy of feature selection by CIFE, JMI, Fisher, Trace Ratio, Lars, HSIC-Lasso, SMNB, CCM and SVFS over 10 independent runs on genomic datasets.

The general superiority of SVFS can be further witnessed on genomics datasets with large number of features as shown in Figure 5. Note again that $Th_{irr}=3$ and $Th_{red=4}$ for all these datasets. However, it is possible to tune the parameters $Th_{irr}$ and $Th_{red}$ to obtain better results per dataset. This can be particularly useful when we focus on specific datasets for disease diagnosis and biomarker discovery.

We conclude from Figures 3, 4, and 5 that our proposed SVFS has achieved the highest accuracy on 12 datasets out of the total 25 datasets, while noting that no other method has achieved the highest accuracy for more than 4 datasets. In cases where SVFS has not produced the highest accuracy, its performance is nonetheless among the most accurate ones.

**Figure 6:**
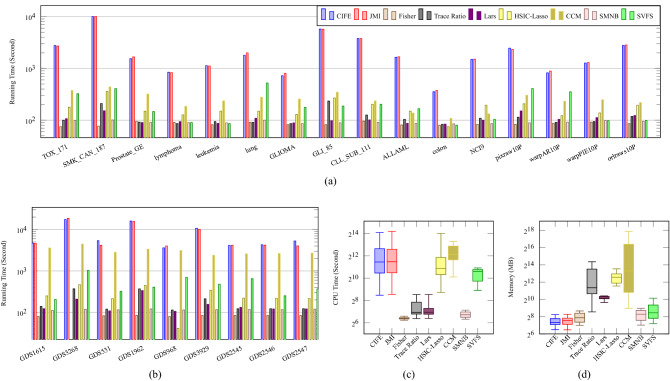
(a), (b) Running Time, (c) CPU Time and (d) Memory taken by CIFE, JMI, Fisher, Trace Ratio, Lars, HSIC-Lasso, CCM, SMNB and SVFS over 10 runs using RF classifier.

Since IBM LSF is capable of reporting running time, CPU time, and memory usage by each feature selection model, we depict the running time in seconds for all feature selection methods in Figure 6. As there are 25 datasets for the evaluation process, Figure 6(a) includes the running time on the benchmark biological and benchmark image datasets and Figure 6(b) covers the running time on the genomic datasets. Note that the reported running times include the RF classification time. It can be seen that the running times of CIFE and JMI are worse than other methods while the running time of CCM method on GEO datasets is high and roughly the same as CIFE and JMI. The other methods including SVFS have comparable and very reasonable running times in the sense that these methods can be comfortably run on regular PCs.

Some methods because of their immense cost of computing are implemented in parallel to perform in reasonable running time. Since HSIC-Lasso hired all available core of CPUs, its CPU time is comparable with CIFE and JMI methods, as shown in Figure 6(c). Moreover, the CCM model takes advantage of TensorFlow^52^ with an optimized CPU implementation in a parallel way, leading to a high CPU time on most of the datasets. The rest of the methods are implemented in a non-parallelized manner; therefore, their CPU times are relatively similar to their running times.

In terms of performance in memory usage, Figure 6(d) shows that CIFE, JMI, Fisher, SMNB, and SVFS are efficient and required comparatively low memory. In contrast, CCM, HSIC-Lasso, and Trace Ratio required a high volume of memory in the magnitude of thousands.

## Conclusion

In this paper, we have proposed a feature selection method (SVFS) based on singular vectors of a matrix. Given a matrix *A* with its pseudo-inverse $A^{\dagger }$, we showed that the signature matrix $S_A=I-A^{\dagger }A$ can be used to determine correlations between columns of *A*. To do this, we associate a graph where the vertices are the columns of *A* and columns ${{\mathbf {F}}}_i$ and ${{\mathbf {F}}}_j$ are connected if $S_{i,j}\ne 0$. We show that connected components of this graph are the clusters of columns of *A* so that columns in a cluster correlate only with columns in the same cluster. We consider a dataset $D=[A\mid \mathbf {b}]$, where rows of *A* are samples, columns of *A* are features, and $\mathbf {b}$ is the class label. Then we use the signature matrix $S_D$ and its associated graph to find the cluster of columns of *D* that correlate with $\mathbf {b}$. This allows us to reduce the size of *A* by filtering out the columns in the other clusters as irrelevant features. In the next step, we use the signature matrix $S_A$ of *A* to partition columns of *A* into clusters and then pick the most important features from each cluster.

A comprehensive assessment on benchmark and genomic datasets shows that the proposed SVFS method outperforms the state-of-the-art feature selection methods. Our algorithm includes two thresholds $Th_{irr}$ and $Th_{red}$ that are used to filter out irrelevant and remove redundant features, respectively. The thresholds have been set the same for all the datasets. However, it is possible to further tune the parameters $Th_{irr}$ and $Th_{red}$ to obtain better results. This can be particularly useful when we focus on specific datasets for disease diagnosis and biomarker discovery.

### Ethics approval

Not applicable.

### Informed consent

Not applicable.

### Use of experimental animals, and human participants

This research did not involve human participants or experimental animals.
